# Supplementary material for: Kinase activity profiling identifies putative downstream targets of cGMP/PKG signaling in inherited retinal neurodegeneration
Source: Cell Death Discov. 2022 Mar 3;8:93. doi: 10.1038/s41420-022-00897-7 (PMC8894370; doi:10.1038/s41420-022-00897-7)
Supplement: Supplementary file 1 — Supplementary figure legend [file 41420_2022_897_MOESM1_ESM.docx]

**Supplementary Figures**

**Figure S1. Heatmap representing the overall serine/threonine kinase activity in retinal explants.
a**) Comparison of protein phosphorylation between *rd1* *vs*. WT retinal explant cultures. **b**) comparison of CN03 treated *vs*. non-treated (NT) *rd1* retinal explant cultures. The phosphorylated peptides are clustered hierarchically as explained in ‘Data Analysis’ section (red=high phosphorylation; yellow=low phosphorylation).

**Figure S2. Bar graph representing the relative signal intensity of individual antibodies in different retina layers.** The relative signal intensity was calculated as the ratio of the average intensity of the single antibody in a single retina layer divided by the negative control. The analysis was performed from retinal cross-sections derived from n=3 P11 WT mice. Mean with ± SD. OS/IS=outer segment/inner segment, ONL=outer nuclear layer, OPL=outer plexiform layer, INL=inner nuclear layer, IPL=inner plexiform layer, GCL=ganglion cell layer.
