# Supplementary figures and images for: Kinase activity profiling identifies putative downstream targets of cGMP/PKG signaling in inherited retinal neurodegeneration
Source: Cell Death Discov. 2022 Mar 3;8:93. doi: 10.1038/s41420-022-00897-7 (PMC8894370; doi:10.1038/s41420-022-00897-7)

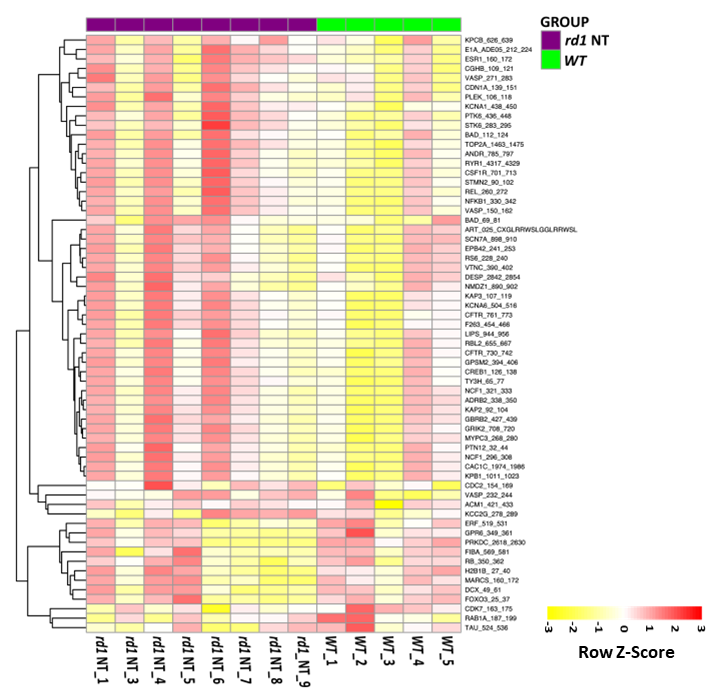

Supplement: Supplementary file 2 — Supplementary Figure 1 [file 41420_2022_897_MOESM2_ESM.tif]

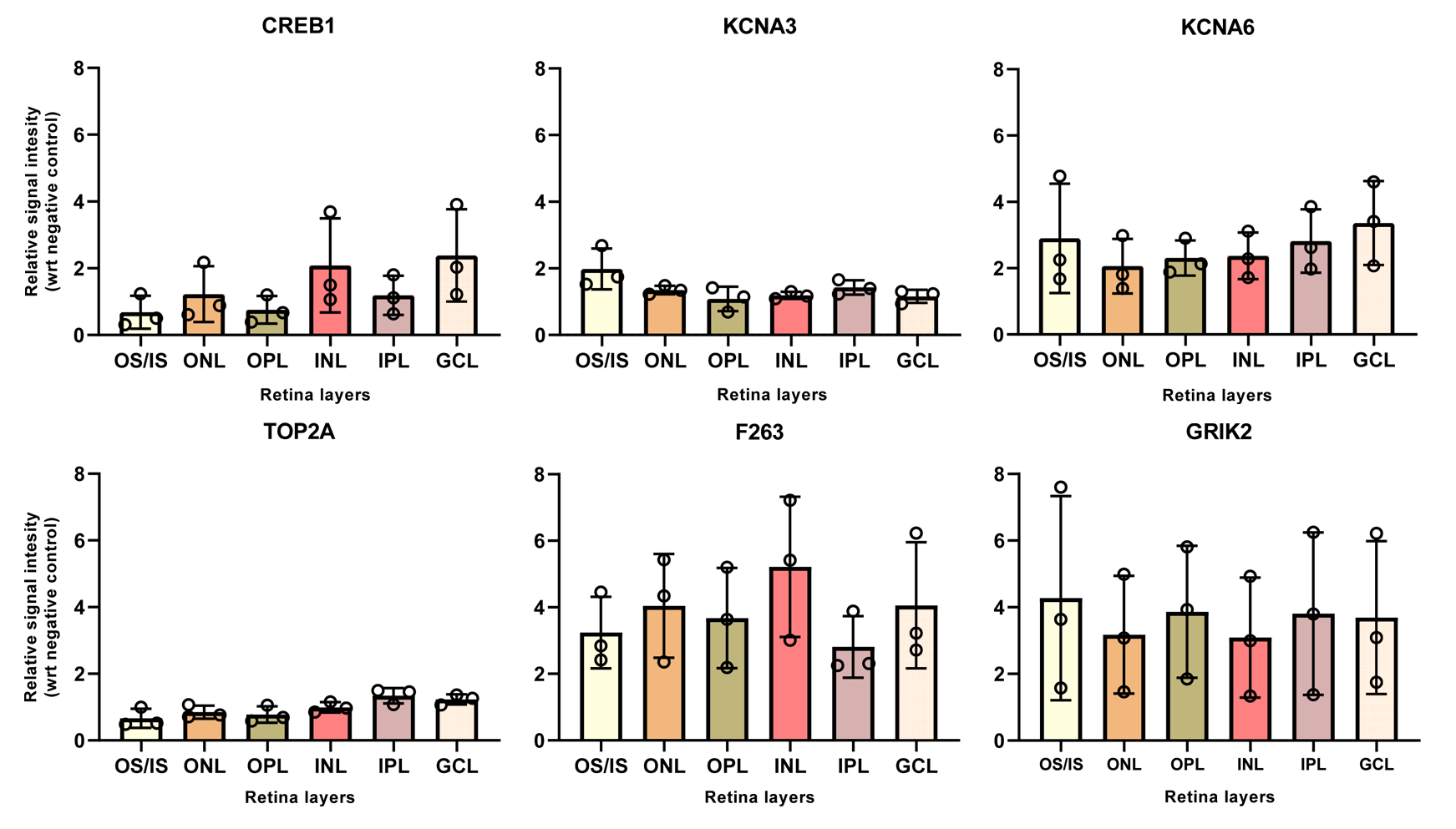

Supplement: Supplementary file 3 — Supplementary Figure 2 [file 41420_2022_897_MOESM3_ESM.tif]
